# Future of Uremic Toxin Management

**DOI:** 10.3390/toxins16110463

**Published:** 2024-10-28

**Authors:** Raymond Vanholder, Evelien Snauwaert, Francis Verbeke, Griet Glorieux

**Affiliations:** 1 Nephrology Section, Department of Internal Medicine and Pediatrics, Ghent University Hospital, 9000 Gent, Belgium; francis.verbeke@uzgent.be (F.V.); griet.glorieux@ugent.be (G.G.); 2 Pediatric Nephrology Section, Department of Internal Medicine and Pediatrics, Ghent University Hospital, 9000 Ghent, Belgium; evelien.snauwaert@uzgent.be; 3 European Reference Network for Rare Kidney Diseases (ERKNet)

**Keywords:** uremic toxins, CKD progression prevention, biotics, senolytics, diet, dialysis, toxin removal, cardiovascular disease in CKD, inflammation, anti-inflammatory treatments, anti-fibrosis

## Abstract

During the progression of chronic kidney disease (CKD), the retention of uremic toxins plays a key role in the development of uremic syndrome. Knowledge about the nature and biological impact of uremic toxins has grown exponentially over the past decades. However, the science on reducing the concentration and effects of uremic toxins has not advanced in parallel. Additionally, the focus has remained for too long on dialysis strategies, which only benefit the small fraction of people with CKD who suffer from advanced kidney disease, whereas uremic toxicity effects are only partially prevented. This article reviews recent research on alternative methods to counteract uremic toxicity, emphasizing options that are also beneficial in the earlier stages of CKD, with a focus on both established methods and approaches which are still under investigation or at the experimental stage. We will consequently discuss the preservation of kidney function, the prevention of cardiovascular damage, gastro-intestinal interventions, including diet and biotics, and pharmacologic interventions. In the final part, we also review alternative options for extracorporeal uremic toxin removal. The future will reveal which of these options are valid for further development and evidence-based assessment, hopefully leading to a more sustainable treatment model for CKD than the current one.

## 1. Introduction

Uremic syndrome, a complex clinical condition that develops when kidney function declines, results in numerous symptoms and complications and accelerated morbidity and mortality [1]. This condition is significantly related to the retention of metabolites that are normally eliminated by healthy kidneys. If these molecules have a negative biological or biochemical impact, they are called uremic toxins. The identification of these retained molecules [2,3] and the unravelling of their biological, biochemical, and clinical impacts [4] coincided with the creation of networks like the European Uremic Toxin Work Group (EUTox) and the growing interest of scientific journals in uremic toxicity.

The study of uremic toxin removal has, until recently, focused on dialysis strategies [2,5]. However, dialysis supports only a small fraction of people with CKD [6,7]. Kidney function replacement by dialysis is far from complete, resulting in a substantially higher mortality risk than in the general population [8,9]. Therefore, there is an urgent need to consider strategies that impact toxin concentrations or their biological effects, not only to improve dialysis strategies but also for earlier stages of chronic kidney disease (CKD). These earlier CKD stages are several-fold more prevalent than advanced CKD requiring kidney replacement therapy (KRT) and even that early show increased morbidity and mortality [8].

Uremic toxins play a crucial role in both mortality and the progression of kidney insufficiency. Comprehensive reviews of the biochemical and clinical impacts of uremic toxins have been published elsewhere [10]. In this publication, we will focus on innovative therapeutic options that could extend the possibilities for reducing the deleterious impact of uremic toxins, particularly in the earlier stages of kidney disease.

## 2. Preservation of Kidney Function 

The preservation of kidney function (Table 1) is the most direct approach to counter uremic toxin retention. Even in individuals treated with dialysis, residual kidney function influences uremic toxin concentration [11], highlighting the crucial importance of preserving kidney function. However, until recently, the prevention of progression of kidney disease was limited to lifestyle interventions and often suboptimal treatments of primary causes like hypertension and diabetes. Currently, several new therapeutic options forestalling CKD progression have become available, like sodium–glucose transporter-2 (SGLT-2) inhibitors [12], non-steroidal mineralocorticoid receptor antagonists [13] (nsMRA), semaglutide [14], and several options for specific rare kidney diseases [15]. In the case of immune-mediated diseases, corticosteroids and other immunosuppressive agents are used, though at the expense of an increased propensity for infection. Additionally, corticosteroids enhance protein catabolism by increasing the availability of nitrogen for urea generation, bone loss, insulin resistance, and the risk of a range of fatal and non-fatal cardiovascular diseases.

Kidney fibrosis plays a central role in the progression of kidney insufficiency, making anti-fibrotic strategies crucial for preserving kidney function [16,17]. Although inflammation and thrombotic events in the kidney may trigger this fibrosis, the underlying processes still remain incompletely understood, and research to find adequate therapeutic solutions is still ongoing [17].

### 2.1. Primarily Anti-Fibrotic Strategies

SGLT-2 inhibition decreases glucose reabsorption in the kidney proximal tubules and is nephroprotective [12] by reducing intraglomerular pressure and improving tubular metabolism and oxygenation [18]. Conversely, SGLT-1 inhibition decreases intestinal glucose absorption and has been shown in a rat adenine model of kidney insufficiency to protect against kidney fibrosis and inflammation, possibly through a change in intestinal microbial uremic toxin production [19].

Pirfenidone is a drug with well-known experimental anti-fibrotic potential [20,21], but its clinical use in CKD has been hampered by dosage concerns, as its removal essentially depends on kidney function. Although an open label study suggested a positive effect on the progression of focal segmental glomerulosclerosis [22], controlled study data are needed to clarify its potential impact.

Pentoxifylline is known for its anti-inflammatory and anti-fibrotic properties [23,24], as well as its capacity to maintain endothelial functional integrity [25]. A meta-analysis showed that pentoxifylline decreased albuminuria but was inconclusive for aggregated eGFR data, although a nephroprotective effect was found in subgroups such as people with more advanced CKD stages and in studies with longer follow-ups [26]. A randomized controlled trial showed that pentoxifylline increased hemoglobin [27]. Although the drug caused no significant change in erythropoiesis stimulating agent administration, the observed 8% decrease was equivalent to a cost-saving of >USD 1100/person in 4 months [27]. A post hoc follow-up study of a randomized controlled trial for up to 7 years showed a nephroprotective effect and a decrease in cardiovascular mortality [28]. There is a need for well-designed long-term hard-outcome studies on this drug.

Targeting transforming growth factor β-1 (TGFβ-1), given its central role in fibrosis, is also a valid option. The effect of leucine-rich alpha-2 glycoprotein-1 [29] (LRG-1) and small interfering RNAs specific to the TGFβ-1 gene [30] have been tested experimentally.

Mesenchymal stem cells have been assessed, showing a positive effect [31]. However, safety concerns have been raised, especially regarding carcinogenicity, the transfer of undesired genetic information, viral contamination, and stem cell rejection [31]. None of the currently evaluated approaches have yet resulted in a clinical breakthrough.

### 2.2. Primarily Anti-Inflammatory Strategies

Inflammation and fibrosis are closely linked, and anti-inflammatory approaches may protect the kidneys. Fibrotic damage due to inflammation occurs through free radical production (oxidative stress), causing activation and proliferation of fibroblasts and myofibroblasts. Nuclear factor erythroid-2-related factor 2 (Nrf2) defends against oxidative damage [32]. Some data suggest a positive effect of bardoxolone on Nrf2 and oxidative stress [32]. Bardoxolone showed promising positive effects on the estimated glomerular filtration rate (eGFR) in diabetes in a phase II trial [33], but a phase III study was halted due to undesirable cardiovascular effects [34]. Further controlled studies on bardoxolone are ongoing.

Other components, like resveratrol and curcumin, are potentially anti-inflammatory and anti-fibrotic, but clinical data in CKD are inconclusive [35,36]. For resveratrol, a bimodal experimental effect was observed, showing anti-fibrotic and pro-fibrotic properties, depending on the concentration [37].

Nicotinamide is another compound with an anti-inflammatory and deoxidating potential, functioning as a precursor of nicotinamide adenine dinucleotide [38] (NAD+). It also restores glycolysis and Krebs cycle activity, thereby neutralizing the harmful effects of insufficient energy procurement causing ischemia. However, nicotinamide may have a dual role as it is also the precursor of the potentially toxic N-methyl-2-pyridone-5-carboxamide [39] (2PY).

Short-chain fatty acids like propionate and butyrate, generated by the gut microbiota, protect kidney function both experimentally and clinically [40,41].

Notably, since many uremic toxins have pro-inflammatory and pro-fibrotic effects, any measure to limit their accumulation also has a potential nephroprotective effect.

In conclusion, preserving kidney function significantly impacts uremic toxin concentrations. After decades of stagnation in strategic approaches, we now have several tools to address kidney function deterioration, and there are more in the pipeline. Further research is needed to expand the possibilities for preserving kidney function, particularly by directly affecting kidney fibrosis, and preferably not only by halting but also by reversing progression, but this ideal solution is to the best of our knowledge rarely achievable at this moment.

### 2.3. Combating Cell Senescence

CKD is characterized by accelerated and premature cell senescence [42], which plays a key role in fibrosis and inflammation [43]. Growing interest in the pathophysiologic role of cell senescence has spurred the search for therapeutic solutions, particularly senolytic drugs.

Experimentally, cell senescence has been addressed through various pathways, using substances like niacin and resveratrol (sirtuin activation), quercetin and fisetin (anti-oxidant flavonoids), panobinostat (a histone deacetylase inhibitor), 2-deoxy-D-glucose (causing cell cycle arrest), and dasatinib (a tyrosine kinase inhibitor) [43,44,45].

Metformin, traditionally used to treat type 2 diabetes, also exhibits strong anti-inflammatory, anti-oxidant, anti-fibrotic, and overall anti-aging effects via the inhibition of nuclear factor (NF)-κB. This results in the attenuation of typical aging diseases like cardiovascular disease, cancer, and diabetes [46]. An RCT evaluating its nephroprotective effects in non-diabetic patients with progressive CKD is ongoing (NCT03831464).

Rapamycin, originally developed as an anti-cancer drug, is a potent senolytic. However, its systemic use is limited by side-effects. Local delivery to the kidneys or nearby tissue, such as through microspheres, could enhance efficacy while minimizing systemic effects [47]. Similar solutions could benefit other senolytics, especially those like several chemotherapeutics and checkpoint inhibitors that affect critical steps in metabolism [43].

Strategies to prevent cell senescence are under intensive study, but clinically validated solutions for CKD are still lacking. Further research is needed, including the repurposing of existing drugs like the antidiabetic metformin and the development of new senolytic therapies.

## 3. Combating Cardiovascular Damage 

Kidney disease and cardiovascular damage are intimately linked [48,49]. Kidney insufficiency induces functional changes that cause cardiovascular damage [50] and the retention of various substances that damage heart and vessels [49,51,52]. Conversely, heart failure and the narrowing of the arterial bed upstream of the kidneys decrease kidney clearance capacity [53]. Hence, the cardiovascular system is affected from the early stages of CKD [8], while the risk of CKD is increased in cardiovascular disease [54].

Minimizing cardiovascular damage (Table 3) is likely to benefit kidney function. In what follows, we will only review kidney-specific mechanisms that may result in useful therapeutic approaches.

### 3.1. Mitigation of Chronic Kidney Disease–Bone and Mineral Disorder (CKD-MBD)

Bone and mineral disorders play a prominent role in cardiovascular damage of CKD and follow a specific course that involves phosphate, fibroblast growth factor 23, parathyroid hormone, sclerostin, and vitamin D [55]. The basic process closely resembles bone formation, with hydroxyapatite as one of the determinants. In a placebo-controlled study on hemodialysis aimed at diminishing the impact of hydroxyapatites, intravenous myo-inositol hexaphosphate decreased vascular wall and aortic valve calcification [56].

One of the other key mechanisms that has emerged in recent years is the generation of circulating calciprotein particles (CPPs) in CKD serum [57], which trigger vessel wall calcification to the point of no return [58] and endothelial dysfunction [59].

Magnesium handling is disturbed in CKD. Higher magnesium concentrations have been associated with decreased vascular calcification [60]. In vitro experiments have suggested this effect is due to the inhibition of the generation of calciprotein particle types with the highest vascular calcification induction rates [61]. Affecting these mechanisms may benefit cardiovascular and kidney conditions, but clinical proof that magnesium correction benefits the cardiovascular status is currently lacking.

### 3.2. Biotics and Cardiovascular Damage

Apart from the contribution of the intestinal microbiome to the generation of uremic toxins, which at the same time are toxic for the cardiovascular system (reviewed in [62]), the intestinal microbiome may also directly impact the cardiovascular system. Also, this mechanism should be thoroughly assessed, especially regarding factors common to both CKD and cardiovascular disease [63,64,65]. Interestingly, while a previous review could not find evidence corroborating toxicity for phenylacetylglutamine [66] (PAG), which also originates from the gut microbiome, PAG was more recently linked to heart failure in both observational clinical studies and mechanistic experimental studies [67]. PAG may become a new target for therapeutic developments.

### 3.3. Anti-Inflammatory Approaches

Inflammation has a strong link with cardiovascular damage. Approaches targeting inflammation have a high potential to benefit cardiovascular status [68,69,70]. Ample evidence indicates that uremic toxins induce cardiovascular damage by promoting inflammation and oxidative stress [52]. Additionally, pro-inflammatory factors contribute to other CKD-specific comorbidities like cachexia [71]. Consequently, any approach that decreases uremic toxin concentration has the potential to decrease cardiovascular risk. Advanced glycation end products (AGEs) have a strong pro-oxidative impact [72], and pharmacologic approaches to circumvent their impact described earlier [72,73], or more recently [74], have to the best of our knowledge only been tested experimentally but not clinically.

A specific link exists between periodontitis, inflammation, and cardiovascular disease [75]. A correlation has been found between dental condition and uremic toxin concentrations in saliva [76], although it is unclear whether this association is coincidental or causative.

In brief (Table 2), preventing cardiovascular damage is an important objective to slow down the progression of kidney insufficiency. Many pathways can be targeted to prevent cardiovascular disease in general, as well as cardiovascular disease induced by CKD-specific pathways. Among these, changes in bone metabolism, magnesium handling, inflammation, periodontitis, the intestinal microbiome, and uremic toxin accumulation are of special interest.

## 4. Gastro-Intestinal Interventions 

Interventions impacting the gastro-intestinal system (Table 3) are crucial as many uremic toxins originate from dietary factors, either directly or through the metabolism of nutrients by the intestinal microbiota [77]. Major protein-bound uremic toxins, such as indoxyl sulfate and p-cresyl sulfate [78,79], as well as small water soluble compounds like trimethylamine-N-oxide [80], are examples of the latter phenomenon. As CKD progresses, the intestinal microbiome shifts significantly to favor bacterial species that produce uremic toxin precursors [81] although increased toxin concentrations in CKD seem largely the result of reduced urinary excretion [82]. The microbiome shift may result from the selection of certain strains and the elimination of others, although the reasons why remain in part speculative. One reason is the common approach to reduce dietary fiber intake to reduce potassium load, which shifts intestinal fermentation in CKD from saccharolytic to proteolytic, although clinician attitudes towards potassium intake are currently changing [83].

### 4.1. Nutritional Interventions

As many toxins originate from proteins, shifting to a low-protein, high-fiber diet reduces uremic toxin production. This diet also provides multiple additional benefits, such as increasing bicarbonate availability mitigating acidosis [10], and delivering an increased potassium load that aids blood pressure regulation and protects kidney function [84]. However, caution is necessary with low-protein diets in dialysis patients due to a high protein need stemming from amino acid losses into the dialysate [85].

A meta-analysis demonstrated that increasing dietary fiber intake can reduce serum levels of indoxyl sulfate, p-cresyl sulfate, blood urea nitrogen, and uric acid [86]. This approach may slow the progression of kidney dysfunction by reducing uremic toxins and inflammation and normalizing intestinal permeability [87]. In children, an inverse relationship was found between fiber intake and levels of free indoxyl sulfate, p-cresyl sulfate, indole acetic acid, and p-cresyl glucuronide [88]. However, a systematic review of five other studies showed inconclusive results regarding fiber’s effect on protein-bound uremic toxin concentrations but consistently found benefits for inflammatory markers [89].

In a study involving subjects with advanced CKD not on KRT, a very-low-protein diet decreased pro-inflammatory intestinal bacteria and restored intestinal wall permeability, compared to standard and Mediterranean diets [90,91]. Both very-low-protein and Mediterranean diets reduced plasma indoxyl sulfate and p-cresyl sulfate levels. However, very-ow-protein diets may increase the risk of malnutrition in CKD, even in non-dialysis patients. Keto-analogues can help sustain low-protein diets while minimizing the risk of malnutrition. Supplementing the Mediterranean diet with keto-analogues modified the intestinal microbiome and decreased indoxyl sulfate and p-cresyl sulfate levels more effectively than the Mediterranean diet alone, but less effectively than a keto-analogue-supplemented very-low-protein diet [92].

A meta-analysis showed a beneficial effect of omega-3 fatty acids on oxidative stress markers, though this conclusion was based on a limited number of studies [93]. Omega-3 fatty acids also attenuated uremic brain damage in mice [94]. However, in inflammatory conditions like CKD and diabetes, omega-3 fatty acids can undergo oxidation, producing 4-hydroxy-2-hexenal, a toxic aldehyde that accumulates in CKD [95] and that might contribute to insulin resistance [96]. An observational study with a 25-year follow-up found an inverse relationship between omega-3 fatty acids and CKD incidence among young American adults [97]. A clinical meta-analysis suggested reduced cardiovascular deaths in hemodialysis patients but showed less convincing results regarding death and kidney failure in CKD patients not on dialysis [98]. An RCT after myocardial infarction showed a minor gain in eGFR of 2.1 mL/min/1.73 m^2^ after 40 months of omega-3 fatty acid treatment compared to placebo, however raising the question whether such a change would be sufficient to impact uremic toxicity [99]. A 5-year follow-up randomized controlled trial on type 2 diabetics showed no impact at all [100]. Finally, it has been suggested that omega-3 supplementation promotes atrial fibrillation [100].

Fecal butyrate exerts a protective effect on the intestinal barrier, and its fecal concentration decreases as kidney dysfunction progresses [62]. Targeting an increase in the population of butyrate-producing bacteria or increased butyrate production by those bacteria, for instance through a very-low-protein diet [91], may be a potential strategy to restore this aspect of intestinal dysbiosis.

### 4.2. Biotics and Uremia

The composition of the intestinal microbiome can also be adjusted by administering biotics, such as prebiotics, probiotics, synbiotics, and postbiotics. Numerous studies have demonstrated the beneficial impact of various biotic combinations on uremic toxin concentrations in serum and feces [101,102,103,104,105,106]. However, the rationale for preferring a specific combination of biotics over others is often not specified. More in-depth insight into the pathophysiological changes of intestinal dysbiosis is likely needed before selecting biotics with an optimal impact.

Several clinical and biochemical benefits of biotics have been suggested, such as improvements in gastrointestinal symptoms [101], cardiovascular status [63,64,107] including vascular aging [65], psycho-cognitive functioning [107], oxidative stress parameters [64], and kidney function deterioration [108,109]. However, confirmation in controlled hard-outcome studies is still lacking.

### 4.3. Intestinal Chelation

The intestinal absorption of uremic toxins can also be impacted by scavenging uremic toxins via sorbent administration. The use of chelators has long been common practice in CKD for phosphate binding [110]. The application of intestinal adsorption of uremic toxins has also become a clinical practice, mostly in Asia [111]. The most classic and most frequently tested options, like AST-120, are derived from activated carbon [110]. Clinically, AST-120 has successfully decreased concentrations of indoxyl sulfate [112], but in controlled studies, it failed to prevent the progression of kidney disease [113,114]. However, in the first study (EPICC), the impact on uremic toxins was not checked [113], whereas in the other, no effect was found on indoxyl sulfate concentrations [114]. A post hoc analysis of the EPICC study in an adherent US subgroup showed a slower progression of kidney disease with AST-120 treatment [115].

### 4.4. Fecal Transplantation

Another option is fecal transplantation, which has primarily been tested in rodents [116]. In mice, this intervention decreased concentration of p-cresol sulfate [117]. However, this strategy imposes safety and ethical issues, especially in cases of less critical indications than those of Clostridioides Difficile infections [118]. At the time of this text’s publication, several studies on fecal transplantation in CKD are ongoing, with one published study, though it lacks data on uremic toxin concentration [119].

In conclusion (Table 3), interventions affecting the intestine as an abundant source of uremic toxins will conceivably influence uremic toxin concentration. Options comprise dietary protein restriction, increased fiber intake, the administration of biotics, and the scavenging of toxins or their precursors with sorbents. Although several of these interventions impact uremic toxin concentration, controlled studies showing a clinical benefit are virtually non-existent.

## 5. Pharmacologic Interventions 

Another option to reduce the biological and clinical impacts of uremic toxins is counteracting metabolic alterations caused by uremic toxins via drug treatment (Table 4). These interventions may not necessarily affect toxin concentration but can diminish toxin activity. Although the pathways that could be impacted are multiple, this option is only at the very early stage of knowledge acquisition.

### 5.1. SGLT-Inhibition

Recent data on SGLT2-inhibitors suggest that, in addition to their effect on the progression of kidney insufficiency [12,120], they also lower uremic toxin concentration directly. After SGLT2-inhibitor administration to healthy individuals, uric acid in serum decreased due to enhanced tubular excretion of uric acid, following increased urinary glucose losses [121]. Other data suggest an impact via intestinal metabolism, causing a decrease in protein-bound uremic toxins, coupled with an increase in fecal short-chain fatty acid concentration, both in a rodent model of CKD [122] and a model of non-diabetic and diabetic mice with normal kidney function [123]. These changes occurred without affecting kidney function. This effect can partly be attributed to a modest inhibitory effect on SGLT-1, favoring intestinal glucose fermentation over protein fermentation [122,124]. Direct SGLT-1 inhibition also decreased serum uremic toxin concentration, which, remarkably, seemingly was mediated by a protective effect on kidney function rather than changes in intestinal microbiome metabolism [19].

### 5.2. Anti-Inflammatory Strategies

Inflammation as a source of damage may be influenced by pharmacologic interventions targeting pro-inflammatory transcription factors [68,125]. Nrf2 activation has an anti-inflammatory impact and is impaired in CKD [32]. It has been hypothesized that Nrf2 activation would inhibit CKD-induced inflammation, potentially retarding oxidative stress, cardiovascular damage, and CKD progression [70]. The promising results with bardoxolone [33], that subsequently could not be confirmed [34], are described above (preserving kidney function).

**Table 4:** Pharmacological interventions.

Overall Group	Therapeutic Strategy	Remarks
SGLT-inhibition	SGLT2-inhibition SGLT1-inhibition	Decreases uremic toxin concentration without an effect on kidney function Decreases uremic toxin concentration more by nephroprotection than by a gastro-intestinal effect
Anti-inflammatory strategies	Nrf2-blockade Cytokine blockade - IL-6 receptor blockade - IL-1 blockade - TNF-α blockade Inhibition of mitochondrial free radical production	Phase III clinical study prematurely terminated due to fatal adverse events Higher risk of infectious complications. Blunted expression of markers of infectious disease Needs further investigation
Blockade of uremic toxin actions	AHR blockage	AHR present in many organs, with also beneficiary roles. May be solved by selectively targeting certain organs or effects
Blockade of uremic toxin production	Structural analogues For TMAO: - DMB - IMC	Only in animal experiments

Abbreviations: SGLT—sodium–glucose transporter, Nrf2—nuclear factor erythroid-2-related factor 2; IL–interleukin; AHR—aryl hydrocarbon receptor; TMAO—trimethylamine-N-oxide; DMB—3,3-dimethyl-1-butanol; IMC—iodomethylcholine.

Another interesting target is the group of pro-inflammatory cytokines. In a meta-analysis, interleukin-6 receptor blockade had a mitigating impact on coronary heart disease [126]. Similarly, interleukin-1 blockade has the potential to decrease cardiovascular risk, as demonstrated in rheumatoid arthritis [127]. In another clinical study, Canakinumab, a monoclonal antibody targeting interleukin-1β, counteracted the progression of atherosclerosis [128]. The interleukin-1 receptor antagonist Anakinra protected against anorexia in an animal model [71].

In human endothelial cells, the TNF-α blocker and Nrf2 inhibitor brusatol reduced endothelial inflammation in vitro and inhibited arteriosclerosis development in two mouse models [129]. Additionally, in a rat model of diabetic nephropathy, TNF-α inhibition reduced glomerular, and tubular damage and albuminuria, [130]. In patients with long-standing severe rheumatoid arthritis, TNF-α blockade had a beneficial effect on endothelial function [131]. One study in subjects with rheumatoid arthritis was suggestive of less cardiovascular events with TNF-α blockade compared with non-biologic disease-modifying anti-rheumatic drugs [132], which was confirmed by a systematic review and meta-analysis [133]. However, we found no controlled hard-outcome studies in kidney disease.

However, the potential benefits of interleukin blockade come at the expense of increasing the risk of infectious complications and the blunted expression of inflammatory markers, delaying infection diagnosis [127]. Despite the clear link between several cytokines and uremic toxicity [66], to our knowledge, no interventional studies have demonstrated a benefit of cytokine blockade in CKD [134].

Finally, mitochondria could be targeted by antioxidants as an anti-inflammatory strategy, because mitochondria are the main organelles to produce oxygen radicals [135].

### 5.3. Blockade of Uremic Toxin Actions

The action mechanisms of specific uremic toxins can also be therapeutically targeted. The aryl hydrocarbon receptor is a central mediator of the toxicity of several uremic toxins derived from tryptophan [136], and inhibiting this receptor could reduce the toxicity of tryptophan-derived metabolites [137]. However, the ubiquitous presence of aryl hydrocarbon receptor in many organs, and its partially beneficial roles [136], makes it challenging to develop appropriate clinical applications, unless specific organs or effects could be targeted.

### 5.4. Blockade of Uremic Toxin Production

A final option is to block the metabolic production of uremic toxins, e.g., by administering structural analogues of uremic toxin precursors. The intestinal generation of the cardiotoxin trimethylamine-N-oxide (TMAO), for which choline and betaine act as precursors [80], was experimentally blocked by administrating 3,3-dimethyl-1-butanol (DMB) in mice [138]. In a model of adenine-fed apolipoprotein E knock-out mice, the administration of the choline analogue iodomethylcholine (IMC) reduced TMAO, improved markers of kidney function, and had a cardioprotective effect [139].

In summary (Table 4), the pharmacological mitigation of uremic toxicity is an insufficiently exploited therapeutic option in CKD. Solutions could aim at diminishing toxin concentration, blocking inflammatory mediators, or directly impacting the metabolic pathways that are impaired by uremic toxins.

## 6. Extracorporeal Toxin Removal 

Finally, in advanced stages of CKD, uremic toxin removal can also be obtained by dialysis and related strategies (Table 5).

### 6.1. Dialysis Strategies

Hemodiafiltration is currently the most frequently applied strategy to enhance the removal of large uremic solutes. After several unsuccessful controlled trials comparing on-line hemodiafiltration to standard hemodialysis, a well-designed randomized study (CONVINCE) showed a survival advantage for hemodiafiltration [140]. In addition, circumstantial evidence from mostly observational studies showed a positive effect on surrogate endpoints such as inflammation, anemia, and neuropathy [141].

However, the results of CONVINCE are in principle only applicable to subjects with the same characteristics as those included in the study, i.e., individuals in whom a high exchange volume (≥23 L per session) can be reached. Additionally, no survival superiority was found in the subgroups with diabetes and cardiovascular disease, and the advantage was only demonstrable for death due to infectious disease, which is a less frequent cause of death in dialysis than cardiovascular disease [142]. It is noteworthy that the study period included the peak years of the COVID-19 pandemic (2020–2021).

The more recently developed medium cut-off (MCO) dialysis membranes allow a safe procedure with a similar “large molecule” toxin removal but without the need for high exchange volumes [143,144], which inspired a redefinition of uremic toxin classification [5]. To the best of our knowledge, there are currently no controlled clinical studies showing outcome superiority or non-inferiority compared to high-volume hemodiafiltration. However, some studies with high-flux hemodialysis as a comparator showed a positive impact on quality-of-life parameters [145]. The difficulties in demonstrating a survival benefit for high-efficiency dialysis strategies may be due to the poor removal of the protein-bound solutes [146], or to the removal of beneficial or even essential molecules that are produced by the same metabolic pathways as several uremic toxins [136].

**Table 5:** Extracorporeal removal.

Overall Group	Therapeutic Strategy	Remarks
Dialysis strategies	Hemodiafiltration Hemodialysis with MCO membranes Peritoneal dialysis	Survival advantage if an exchange volume of >23 L per session can be achieved. No proven survival advantage in DM and CVD Removal capacity similar to hemodiafiltration without need for large exchange volume. No controlled survival data. Circumstantial evidence of positive impact on quality of life Survival comparable to dialysis
Protein-bound toxin removal	Adsorption Fractionated plasma separation and sorbent purification Modification of plasma pH Addition of liposomes to dialysate Infusion of binding competitors - Free fatty acids - Tryptophan - Ibuprofen	No hard-outcome data Increased risk for hypercoagulability Safety concerns Concerns about large scale feasibility Safety concerns
Preserving kidney function	Various strategies (see Table 1)	Presumable effect on toxin concentration even in dialysis. No hard clinical data
Green dialysis concept	Decrease energy consumption (greenhouse gas emissions) Decrease water consumption (water waste) Decrease plastic waste	Need for more research and development. Need for registration and benchmarking

Abbreviations: DM—diabetes mellitus; CVD—cardiovascular disease; MCO—medium cut-off.

Interestingly, peritoneal dialysis (PD) offers a less efficient removal of uremic toxins than hemodialysis [147], although outcome studies suggest similar survival outcomes for both [148]. This may be partly due to the continuous application of PD or differences in toxin generation. 

Research is ongoing to develop options for the continuous use of implantable or wearable hemodialysis devices, but those still need clinical refinement [149,150].

### 6.2. Protein-Bound Toxin Removal

Several studies have analyzed approaches that enhance the removal of protein-bound molecules. The most successful removal strategy for the time being seems to be adsorption, either by resins [151] or by carbon-based derivatives [152]. Fractionated plasma separation with sorbent purification and plasma reinfusion efficiently removed protein-bound uremic toxins in dialysis patients [153], but an earlier clinical study had found a higher propensity for hypercoagulation with this approach [154]. In vitro, the induction of changes in plasma pH to extremely low or high values markedly increased the free fraction of protein-bound molecules, enabling more diffusive removal [155], but in vivo feasibility remains uncertain. Another option is adding liposomes to the dialysate. In addition, experimental intravenous infusion in rats released protein-bound solutes from their binding sites [156]. In vitro, the spiking with liposomes of human albumin solutions containing protein-bound solutes increased their removal through ultrafiltration [156]. However, questions arise about large-scale applicability [157], due to the complexity of the manufacturing process and difficulties in achieving consistency in the shape and composition of the liposomes [158,159]. In addition, the shelf life is short, the risk of degradation significant [160], and the quantities required to increase the removal of protein-bound uremic toxins are substantial [160], while costs remain high [161]. We are not aware of any clinical studies with this option.

Others have assessed the effect of binding competitors, also to liberate solutes for diffusion [162]. In vitro or ex vivo tests have been performed with free fatty acids, tryptophan, and ibuprofen. However, ibuprofen is less suited due to its potential toxicity and side-effects [162].

### 6.3. Preserving Kidney Function

Of note, even in dialysis, uremic toxin removal is significantly impacted by residual kidney function [11,163,164,165,166], which can be preserved as summarized earlier in this text and in Table 2. However, no data are currently available on a possible outcome advantage of nephroprotective pharmacologic interventions in dialysis. Additionally, a stepwise increase in the number of hemodialysis sessions per week after the initiation of dialysis (incremental hemodialysis) may benefit residual kidney function [164,167].

### 6.4. The Green Dialysis Concept

A largely unsolved burden of dialysis treatment is the environmental impact, contributing to the production of greenhouse gas emissions, water consumption, and plastic waste. Innovative solutions are urgently needed to address these issues [168,169,170]. Although environmental problems related to dialysis can be approached in many ways, the most important parameters to assess per unit, cluster of units, or region are energy consumption, volume of spent water, and material waste. However, this requires careful documentation, benchmarking for comparison with other entities, and the exchange of best practices.

In brief (Table 5), the extracorporeal removal of uremic toxins remains currently limited to variants of the classical dialysis methods. Innovative solutions are needed to better remove protein-bound toxins, preserve residual kidney function, and make dialysis more environment-friendly

## 7. Conclusions

**Figure 1:**
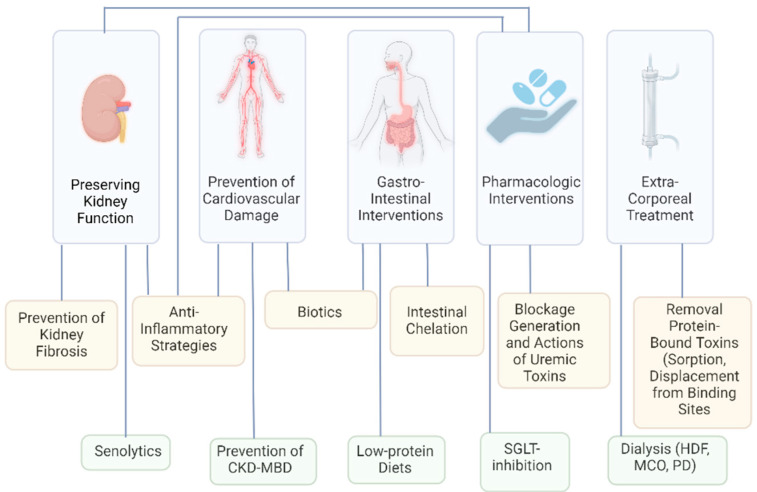
Fields of uremic toxin research that may lead to a therapeutic shift in paradigm. Several fields interfere with each other. Abbreviations: CKD-MBD—chronic kidney disease–metabolic bone disorder; SGLT—sodium–glucose transporter; HDF—hemodiafiltration; MCO—medium cut-off; PD—peritoneal dialysis. Created with BioRender.com.

The basic principles of uremic toxin removal have remained the same for many years. However, increasing knowledge about the granularity of uremic toxicity and the multiplicity of uremic toxins and their origin has fostered numerous studies assessing alternative solutions to the classical hemodialysis concept. This article reviews several of these alternatives (Figure 1), such as approaches to preserve kidney function, change intestinal metabolism, prevent cardiovascular damage, and mitigate biological changes induced by uremic retention. Also, innovative alternative extracorporeal removal strategies are discussed. The future will show which solutions are promising enough for evidence-based testing or clinical application. The current therapeutic model of uremic toxin handling has a low level of long-term economic, environmental, and quality-of-life sustainability. The continued and incremental investment in kidney care research and innovation is needed, proportionally to the disease burden, to allow a more sustainable therapy model to benefit all those affected by CKD.

## Figures and Tables

**Table 1:** Preserving kidney function.

Overall Group	Therapeutic Strategy	Remarks
Primarily anti-fibrotic strategies	SGLT-2 inhibition SGLT-1 inhibition Pirfenidone Pentoxifylline TGFβ-1 inhibition - LRG-1 - Small RNAs interfering with TGFβ-1 Mesenchymal stem cells	Evidence-based nephroprotection Via impact on intestinal microbiome Dependent on kidney clearance. Need for controlled clinical data Controlled long-term hard-outcome studies needed Concerns about carcinogenicity, transfer of genetic information, viral contamination, stem cell rejection
Primarily anti-inflammatory strategies	Bardoxolone Resveratrol Curcumin Nicotinamide Short chain free fatty acids - Propionate - Butyrate	Increase in proteinuria; Nrf2 activation Phase III study halted because of cardiovascular complications Clinical data inconclusive. Experimentally bimodal effect depending on concentration Clinical data inconclusive Potential precursor of 2PY
Senolytics	Niacin, Resveratrol Quercetin, Fisetin Panobinostat 2-deoxy D glucose Destinib Metformin Rapamycin Checkpoint inhibitors Other chemotherapeutics	Sirtuin activation Anti-oxidant flavonoid Histone deacetylase inhibitor Cell cycle arrest Tyrosine kinase inhibitor Inhibition of NF-κB Systemic administration causes complications. Problem may be solved by local delivery Systemic administration causes complications. Problem may be solved by local delivery Systemic administration causes complications. Problem may be solved by local delivery

Abbreviations: SGLT—sodium–glucose transporter; TGFβ-1—tissue growth factor β-1; LRG-1—leucine-rich alpha-2 glycoprotein-1; Nrf2—nuclear factor erythroid-2-related factor 2; 2PY—N-methyl-2-pyridone-5-carboxamide2; NF-κB—nuclear factor kappa-light-chain-enhancer of activated B cells.

**Table 2:** Combating cardiovascular damage.

Overall Group	Therapeutic Strategy	Remarks
Correction of CKD–MBD	Antagonism of vascular hydroxyapatite formation (myoinositol hexaphosphate) Correction of magnesium handling disturbances	Decreased cardiovascular calcification in clinical study Most clinical studies are observational. Interventional controlled studies have been unsuccessful up until now
Biotics	Especially those biotics impacting simultaneously CVD and CKD should be considered PAG antagonism	Experimental and clinical studies suggest a pathophysiological role
Anti-inflammatory approaches	Antagonism of AGEs Dental care (combating periodontitis)	No clinical evidence

Abbreviations: CKD-MBD—chronic kidney disease–metabolic bone disorder; CVD—cardiovascular disease; CKD—chronic kidney disease; PAG—phenylacetylglutamine; AGEs—advanced glycation end products.

**Table 3:** Gastro-intestinal interventions.

Overall Group	Therapeutic Strategy	Remarks
Nutritional intervention	Low-protein, high-fiber diet Very low-protein diet Very low-protein diet plus keto-analogues Mediterranean diet Omega-3 fatty acids	Reduces metabolic acidosis Increases potassium load - Regulates blood pressure - Protects kidney function Risk of malnutrition and hyperkalemia, particularly in dialysis patients Risk of malnutrition in all CKDs Reduces malnutrition risk No compelling evidence of outcome benefit
Biotics	Various combinations	Rationale for chosen combination is often unclear. No hard-outcome data in controlled studies
Intestinal chelation	Activated carbon (AST-120)	Hard-outcome studies inconclusive
Other	Fecal transplantation	Ethical and legal concerns for non-critical indications

## Data Availability

No new data were created or analyzed in this study. Data sharing is not applicable to this article.
